# Clinical and whole exome sequencing findings in children from Yunnan Yi minority ethnic group with retinitis pigmentosa: two case reports

**DOI:** 10.1186/s13256-023-03830-3

**Published:** 2023-06-02

**Authors:** Yi-shuang Xiao, Wen-Ji He, Hong-chao Jiang, Li Tan, Jing Ma, Zhen Zhang

**Affiliations:** 1 Department of Ophthalmology, Kunming Children’s Hospital, Kunming Medical University, Kunming, 650228 Yunnan People’s Republic of China; 2 Kunming Children’s Hospital, Kunming Medical University, Kunming, 650228 Yunnan People’s Republic of China; 3 Department of Otolaryngology-Head and Neck Surgery, Kunming Children’s Hospital, Kunming Medical University, Kunming, 650228 Yunnan People’s Republic of China; 4 Department of Clinical Pharmacy, Affiliated Hospital of Yunnan University, Kunming, 650021 Yunnan China

**Keywords:** Mutation, Retinitis pigmentosa, Pediatric population, Yunnan

## Abstract

**Background:**

Retinitis pigmentosa is a group of rare hereditary retinal dystrophy diseases that lead to difficulty seeing at night, progressive loss of peripheral field vision (tunnel vision), and eventual loss of central vision. However, a genetic cause cannot be determined in approximately 60% of cases.

**Case presentation:**

Two non-consanguineous Yi minority ethnic group families who have a 6.4-year-old boy and a 0.5-year-old boy, respectively, were recruited for genetic diagnosis. Here, we used whole-exome sequencing to detect mutations in the genes of the probands of the retinitis pigmentosa families, and Sanger sequencing to confirm the causal mutations identified by whole exome sequencing. In addition, we report two cases with retinitis pigmentosa caused by *RDH12* (c.524C > T) and *PRPF4* (c.1273G > A) pathogenic mutations.

**Conclusions:**

These results might extend the mutation spectrum of known retinitis pigmentosa genes and give these two Yi minority ethnic group families from Yunnan more precise genetic counseling and more specific prognoses.

**Supplementary Information:**

The online version contains supplementary material available at 10.1186/s13256-023-03830-3.

## Background

Retinitis pigmentosa (RP, MIM# 268000) is a rare inherited retinal disease, and it causes severe vision impairment due to progressive degeneration of the rod photoreceptor cells in the retina [1]. It might cause photoreceptor cells and RP epithelial cells to be damaged and usually presents a variety of symptoms, including night blindness, decreasing visual fields leading to tunnel vision, and total blindness in some instances [2]. An ophthalmologist in the Netherlands, Franciscus Cornelius, first described RP in 1857 [3]. According to the clinical manifestations, there are two main groups of RP, typical RP or rod-cone dystrophy (RCD), in which the rods are predominantly damaged and which represents 80–90% of total cases, and atypical RP or cone-rod dystrophy (CRD), in which the cones are primarily injured and which represents 10–20% of total cases [4]. According to the various types of pigmentary retinopathies associated with many systemic disorders, RP can be expressed in syndromic and non-syndromic forms [5]. The non-syndromic form occurs alone without any other clinical findings, whereas the syndromic form occurs with other neurosensory disorders, developmental abnormalities, or complex clinical findings [6]. The non-syndromic form accounts for approximately 65% of RP cases [7]. RP prevalence is variable among different ethnic groups, with 1 case per 3000–5000 individuals. The total prevalence of RP is 1:4000 in Europe, 1:3500 in the USA, and 1:3784 in China [6, 8–10].

Inheritance patterns of RP are autosomal dominant (adRP), autosomal recessive (arRP), X-linked (XLRP), and mitochondrial transmission. These patterns are dependent on the specific RP gene mutations present in the parental generation.

*RP1* was the first identified genetic cause of RP in 1999 in *Nature Genetics*. Over the last 19 years, nearly 100 different genes have been linked to RP, including 27 adRP genes, 58 arRP genes, and 3 XLRP genes [11]. Although the genetic cloning research for RP has achieved remarkable results and nearly 100 genes of RP have been found in patients, 50% of patients with RP still have unexplained genetic causes. [12] Morphological, clinical, and genetic RP assessments indicate complicated ocular impairment, and RP is known as a “fever of unknown origin.”

In the case of the Chinese population, limited studies are available [13], and there is little known about RP in pediatric populations, especially for Yunnan pediatric individuals with RP due to the fact that various populations in Yunnan are relatively “enclosed” in certain areas. These “enclosed” populations have gradually developed into different ethnic groups because Yunnan mountainous areas lead to poor transportation.

Here, we identified and reported the mutations in two Yi minority ethnic group families from Yunnan, which has been inhabited by 26 different ethnicities throughout history.

## Case presentation

### Clinical diagnosis and sample preparation

Two non-consanguineous Yi minority ethnic group families from the Yun–Gui Plateau were recruited by the Children’s Hospital of Kunming Medical University for genetic diagnosis.

The clinical diagnosis of RP was established by ophthalmological examination in Kunming Children’s Hospital, University of Kunming Medical. Pedigrees were established on the basis of interviews with the patient’s parents. Additionally, in the present study, normal control was 40 individuals aged between 4 and 14 years old, including 20 males and 20 females without associated hereditary diseases.

This study was approved by the Ethics Committee of the Children’s Hospital, University of Kunming Medical, and written consent forms were obtained from participants or guardians. Moreover, written informed consents were obtained from parents of all participants under the age of 16.

Two-milliliter peripheral blood samples from all patients and family members were collected according to the principles of the Declaration of Helsinki, and these samples were collected in tubes containing 0.2 M EDTA. DNA from the peripheral blood samples was extracted using the QIAamp DNA blood extraction kit (Qiagen, Beijing Kangwei Century Biotechnology Co., LTD., Beijing, China). All samples were checked with Nanodrop 2000 or Qubit to determine if they represented a qualified captured library. Then, resulting qualified captured library was loaded on a Illumina Nextseq 500 sequencing platform, and sequencing was conducted to ensure that each sample met the desired average sequencing coverage.

## Case reports

**Fig. 1:**
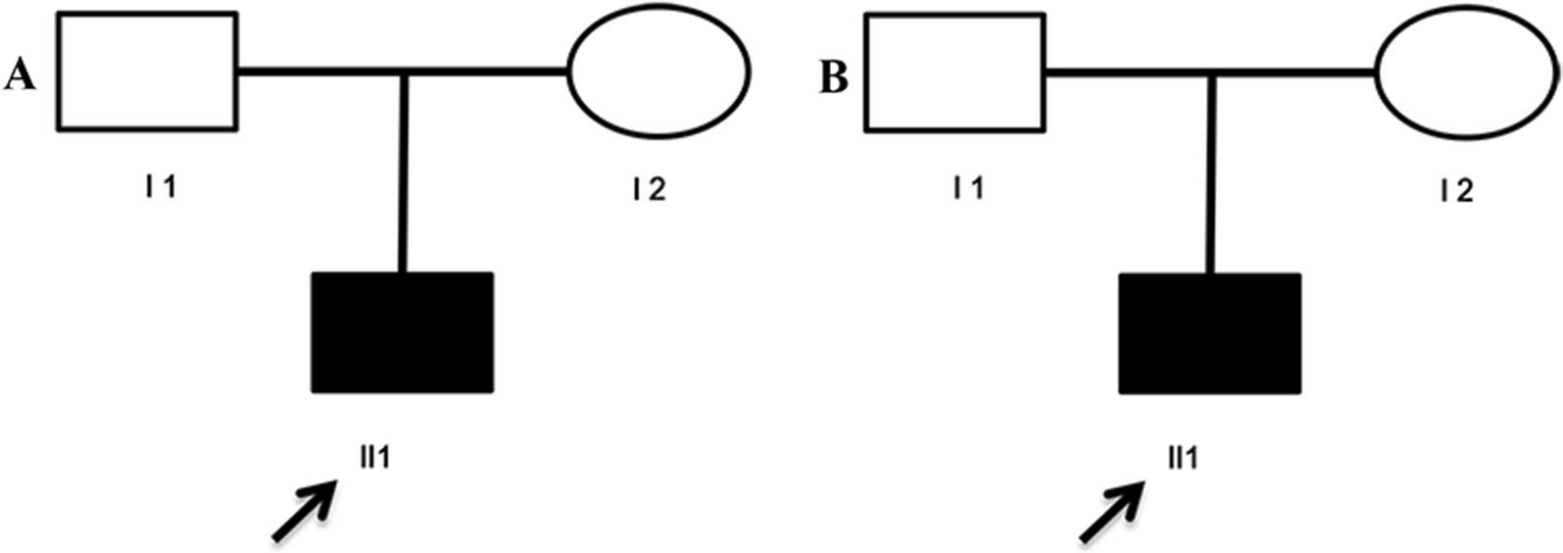
Pedigrees of family. **A** Pedigree of family 1 with RP. **B** Pedigree of family 2 with RP. Unaffected subjects are denoted as blank, while affected subjects are represented with darkened symbols. Arrow indicates the proband

**Fig. 2:**
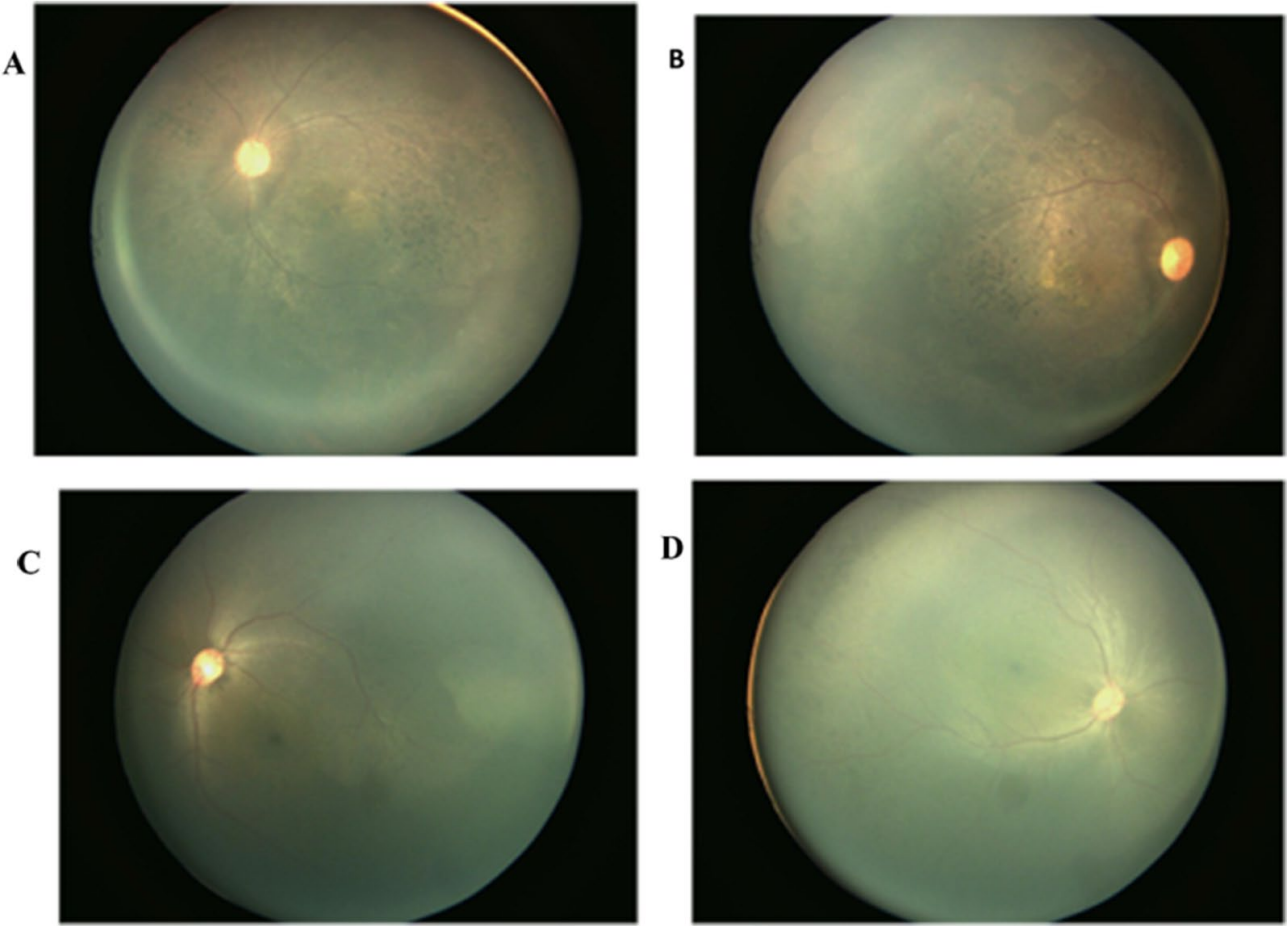
Fundus photographs. **A** and **B** show typical changes of fundus in the left eye and right eye in family 1, respectively. **C** and **D** show typical changes of fundus in the left eye and right eye in family 2, respectively

The first case is of a 6.4-year-old boy who is the only child from non-related parents (Fig. 1). He was born at 39 weeks of gestation without asphyxia by spontaneous vaginal delivery to healthy Yi parents. His birth weight was 2.9 kg. According to his parent’s representation, the proband had a loss of night vision at the age of 5 years old. He was then referred to ophthalmic examination for his poor night vision at 5.8 years old: his cornea was clear, anterior chamber depth was normal, aqueous humor was clean, pupil was round, and reflective light reaction normal. His fundus photography indicated pigmentation around bone cells, narrowing retinal vascular, retinal pigment disorderly distributed, and unclear macular foval reflex (Fig. 2).

The second case is of a 0.5-year-old boy who is the only child from non-related parents (Fig. 1). He was born at 40 weeks of gestation without asphyxia by spontaneous vaginal delivery to healthy Yi parents. His birth weight was 3.1 kg. According to his parent’s representation, the proband had vision problems at the age of 1 month when he had a routine neonatal eye examination. He arrived to our attention after an initial diagnosis was formulated at the age of 4 months. The results of his mobility and blinking activity are presented in Table 1. His fundus photography indicated pigmentation around bone cells and macula foveal reflex was clear (Fig. 2). The detailed clinical data for these two families are presented in Table 1.

**Table 1:** Clinical features of patients with RP

Patient	Age (years)	Sex	Onset age (years)	Vision (refraction)		Fundus appearance	
				LE	RE	LE	RE
1	6.4	M	5.8	0.5 (−1.5DS)	0.6 (−2.0DS)	PD	PD
2	0.5	M	0.3	−2.50DS	−1.5DS	PD	PD

*M* male, *LE* left eye, *RE* right eye, *PD* pigment deposits

## Mutation analysis using whole exome sequencing (WES)

WES was conducted on the two probands of these families. The mean read depth of the target regions of each proband sample ranged from 52–66X, and the average throughput depth of the target region in each sample ranged from 90–99X. Raw image files were processed for base calling and raw data generation with Bcl2Fastq software (Bcl2Fastq 2.18.0.12, Illumina, Inc.). In addition, low-quality variations were filtered out to get a quality score ≥ 20.Then, using Short Oligonucleotide Analysis Package (SOAP) aligner software (SOAP2.21, soap.genomics.org.cn/soapsnp.html), the clean reads were aligned to the reference human genome (UCSC hg19, http://genome.ucsc.edu/). Polymerase chain reaction (PCR) duplicates were removed by the Picard program. The single nucleotide polymorphisms (SNPs) were determined by the SOAPsnp programme. The reads were realigned by Burrows–Wheeler Aligner (BWA) software 0.7.15, and the deletions and insertions (indels) were detected by Genome Analysis Toolkit software 3.7. In addition, the identified indel SNPs were annotated with the Exome-assistant program (http://122.228.158.106/exomeassistant). To determine their pathogenicity, non-synonymous variants were evaluated by four algorithms, namely, PolyPhen (http://genetics.bwh.harvard.edu/pph2/), Protein Analysis Through Evolutionary Relationships (PANTHER, www.pantherdb.org), Sorting Intolerant from Tolerant [SIFT, (http://sift.jcvi.org/)] and Pathogenic Mutation Prediction (Pmut; http://mmb.pcb.ub.es/PMut/).

Compared with the variants in the SNP databases, the Consensus Coding Sequence (CCDS), NCBI 36.3, dbSNP (v138), and the 1000 Genomes Project, two mutations were identified in these two families with RP. There is one mutation in gene *RDH12* in family 1 and *PRPF4* in family 2, respectively (Table 2).

**Table 2:** Mutations identified in these families

Family	Gene	Exon	Nucleotide mutations	Protein effect	Db SNP ID	Mutation type
1	*RDH12*	7	c.524C > T	p.S175L	Known	Non-synonymous
2	*PRPF4*	13	c.1273G > A	p.G425S	Known	Non-synonymous

**Fig. 3:**
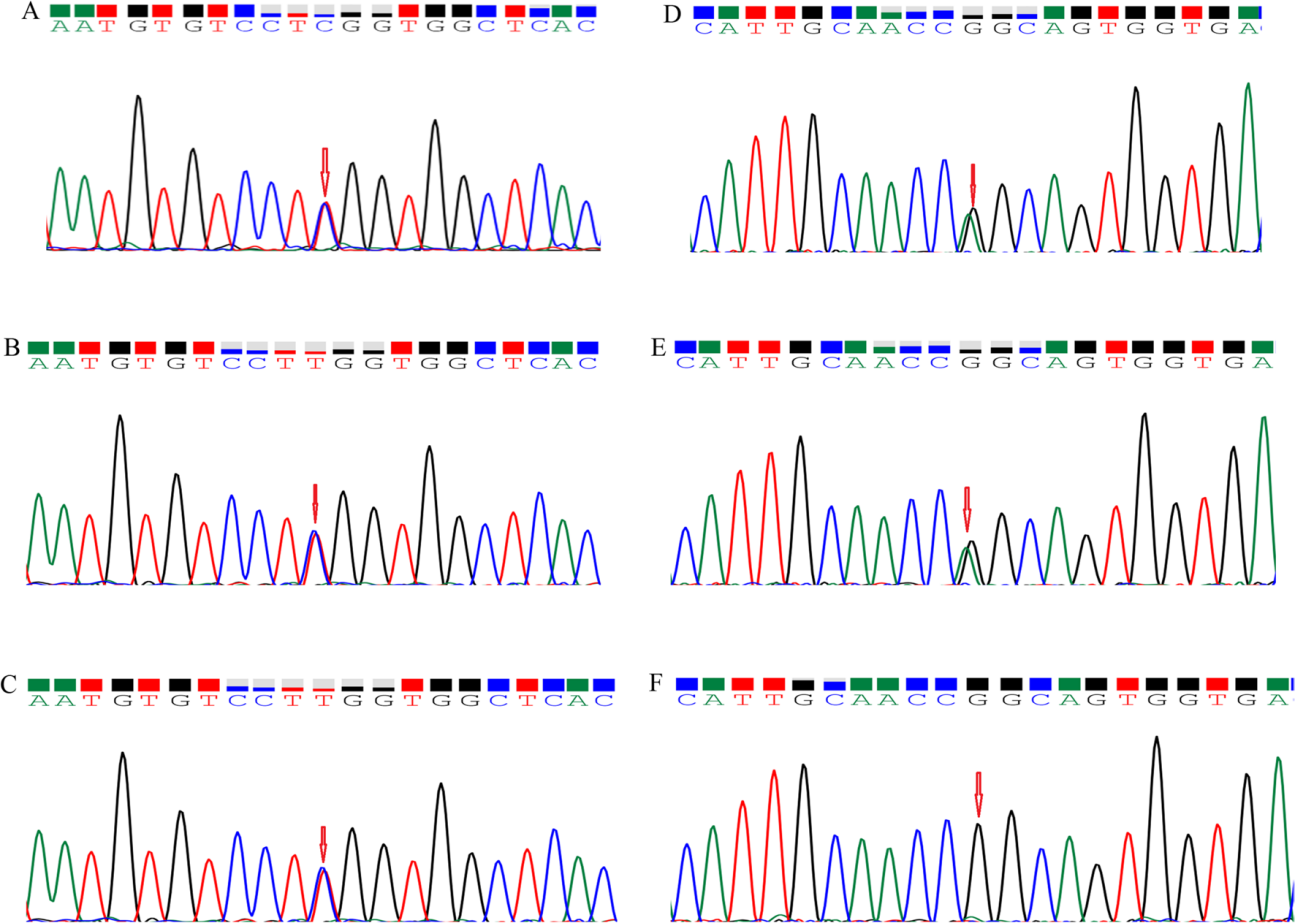
Mutations identified by Sanger sequencing analysis in families 1 and 2. **A** *RDH12* c.524C > T variant of the proband in family 1. Arrows denote the mutations. **B** The unaffected father in family 1. **C** The unaffected mother in family 1. **D** *PRPF4* c.1273G > A variant of the proband in family 2. Arrows denote the mutations. **E** The unaffected father in family 2. **F** The unaffected mother in family 2

Polymerase chain reaction (PCR) and Sanger sequencing with an ABI3500 sequencer were conducted to validate these mutations in these family members to confirm the accuracy of the mutations identified by whole exome sequencing (WES). The sites of variation were identified to compare the DNA sequences with the corresponding GenBank (www.ncbi.nlm.nih.gov) reference sequences. The sequences of forward and reverse primers are presented in Additional file 1: Table S1 and were used to confirm potential causative variants in this family. Thermocycling conditions: an initial denaturation of 95 °C for 10 minutes, 35 cycles of denaturation at 94 °C for 30 seconds, annealing at 64 °C for 30 seconds, extension at 72 °C for 45 seconds, and a final extension of 72 °C for 5 minutes. The results of Sanger sequencing showed that parents were carriers of c.524C > T (p.S175L) (Fig. 3) and mother was carrier of c.1273G > A (p.G425S) (Fig. 3) in family 1 and in family 2, respectively. Additionally, these two variants were absent in 40 normal control individuals.

## Discussion and conclusions

Molecular diagnosis is essential for accurate clinical diagnosis and to provide more precise genetic counseling [14]. To gain insight into the genetic basis of Yunnan pediatric population with RP, which had not been previously studied, two Yi families with RP in Yunnan were recruited and we reported that a WES method was used for the molecular diagnosis of Yunnan children with RP.

In this study, we used WES as an effective tool with potential to improve diagnoses. In addition, we found mutations in two rare genes of RP (The causative genes included two categories: most prevalent genes and rare genes [12]), and are described as follows.

We found mutation c.524C > T in *RDH12* in family 1. The c.524C > T mutation in exon 7 was identified in our study. *RDH12* is a member of the reductase superfamily/short-chain dehydrogenase of proteins that are expressed in photoreceptor cells [15]. The *RDH12* gene encodes a protein composed of 316 amino acids and has a pivotal role in the formation of 11-cis retinal from 11-cis retinol during the regeneration of visual pigments [16]. The mutations of *RDH12* have been identified in patients diagnosed with arRP and adRP [17, 18]. Generally, *RDH12* retinopathy starts at 2–4 years with impaired visual function [19], but the mutation was found in this proband when retinopathy started at 5.8 years.

In family 2, we discovered by WES and Sanger sequencing that the genetic defects were located in *PRPF4*. The mutations c.1273G > A (p.G425S, mother) in *PRPF4* are causative mutations for family 2. The mutations of *PRPF4* result in RP due to the fact that it is a key component of spliceosome, which plays critical roles in pre-mRNA splicing.[20] Up to now, four human heterozygous *PRPF4* variants, p.P187A, p.R192H, c.-114_-97del and c.C944T, have been reported to cause retinitis pigmentosa. [21–23] Among yeast, *Caenorhabditis elegans*, zebrafish, and humans, *PRPF4* is highly conserved.[24] The overexpression of human *PRPF4*^*C944T*^ variant or knockdown of *prpf4* showed a functional conservation between human and zebrafish Prpf4 proteins. Mutations in *PRPF4* cause adRP due to highly conserved C-terminal region of PRPF4, which interacts with pre-mRNA processing factor 3 [21, 22].

Retinitis pigmentosa causes irreversible blindness, and there is no universal cure for RP [12]. So, our follow-up visits and prognosis and proper management of the disease are missing.

This study is limited by the small number of probands, and further studies are necessary to ascertain the impact of these two mutations on gene functions. It is important to establish a molecular diagnosis, which is an essential prerequisite to identifying causative pathogenic variations and a better understanding of the disease. [25]

Our results might extend the mutation spectrum of known *RP* genes in the Yunnan pediatric population. Molecular diagnosis might give these two families more precise genetic counseling and more specific prognoses due to better definitions of inheritance pattern.

## Supplementary Information

**Additional file 1: Table S1.** The primer sequences.

## Data Availability

The analyzed datasets generated during the study are available from the corresponding authors upon reasonable request.

## Abbreviations

RP: Retinitis pigmentosa
CCDS: Consensus coding sequence
RCD: Rod-cone dystrophy
adRP: Autosomal dominant
arRP: Autosomal recessive
XLRP: X-linked

## References

[1] Hartong DT, Berson EL, Dryja TP (2006). Retinitis pigmentosa. Lancet.

[2] Fernandez-San JP (2006). Targeted next-generation sequencing improves the diagnosis of autosomal dominant retinitis pigmentosa in Spanish patients. Invest Ophthalmol Vis Sci.

[3] O'Neal TB, Luther EE. Retinitis pigmentosa. 2023. 30137803

[4] Parmeggiani F, Sato G, De Nadai K, Romano MR, Binotto A, Costagliola C (2011). Clinical and rehabilitative management of retinitis pigmentosa: up-to-date. Curr Genomics.

[5] Costa KA, Salles MV, Whitebirch C, Chiang J, Sallum J (2017). Gene panel sequencing in Brazilian patients with retinitis pigmentosa. Int J Retina Vitreous.

[6] Daiger SP, Sullivan LS, Bowne SJ (2013). Genes and mutations causing retinitis pigmentosa. Clin Genet.

[7] Daiger SP, Bowne SJ, Sullivan LS (2007). Perspective on genes and mutations causing retinitis pigmentosa. Arch Ophthalmol.

[8] Retinitis pigmentosa. A symposium on terminology and methods of examination. Ophthalmology 1983;90(2):126–31. 6856249

[9] Yang L (2015). Dependable and efficient clinical molecular diagnosis of Chinese RP patient with targeted exon sequencing. PLoS One.

[10] Fu Q (2013). Next-generation sequencing-based molecular diagnosis of a Chinese patient cohort with autosomal recessive retinitis pigmentosa. Invest Ophthalmol Vis Sci.

[11] Mercado CL, Pham BH, Beres S, Marmor MF, Lambert SR (2018). Unilateral retinitis pigmentosa in children. J Am Assoc Pediatr Ophthalmol Strabismus.

[12] Bhardwaj A, Yadav A, Yadav M, Tanwar M (2022). Genetic dissection of non-syndromic retinitis pigmentosa. Indian J Ophthalmol.

[13] Haim M (2002). Epidemiology of retinitis pigmentosa in Denmark. Acta Ophthalmol Scand Suppl.

[14] Gonzalez-del PM (2011). Mutation screening of multiple genes in Spanish patients with autosomal recessive retinitis pigmentosa by targeted resequencing. PLoS One.

[15] Valverde D, Pereiro I, Vallespín E, Ayuso C, Borrego S, Baiget M (2009). Complexity of phenotype-genotype correlations in Spanish patients with RDH12 mutations. Invest Ophthomol Vis Sci.

[16] Haeseleer F, Jang GF, Imanishi Y, Driessen C, Matsumura M, Nelson PS, Palczewski K (2002). Dual-substrate specificity short chain retinol dehydrogenases from the vertebrate retina. J Biol Chem.

[17] Beheshtian M, Saee RS, Babanejad M, Mohseni M, Hashemi H, Eshghabadi A, Hajizadeh F, Akbari MR, Kahrizi K, Riazi EM (2015). Impact of whole exome sequencing among Iranian patients with autosomal recessive retinitis pigmentosa. Arch Iran Med.

[18] Fingert JH, Oh K, Chung M, Scheetz TE, Andorf JL, Johnson RM, Sheffield VC, Stone EM (2008). Association of a novel mutation in the retinol dehydrogenase 12 (RDH12) gene with autosomal dominant retinitis pigmentosa. Arch Opthalmol..

[19] Zou X, Fu Q, Fang S, Li H, Ge Z, Yang L, Xu M, Sun Z, Li H, Li Y (2018). Phenotypic variability of recessive RDH12-associated retinal dystrophy. Retina.

[20] Wang Y, Han Y, Xu P, Ding S, Li G, Jin H, Meng Y, Meng A, Jia S (2018). prpf4 is essential for cell survival and posterior lateral line primordium migration in zebrafish. J Genet Genomics.

[21] Chen X, Liu Y, Sheng X, Tam PO, Zhao K, Chen X, Rong W, Liu Y, Liu X, Pan X (2014). PRPF4 mutations cause autosomal dominant retinitis pigmentosa. Hum Mol Genet.

[22] Linder B, Hirmer A, Gal A, Rüther K, Bolz HJR, Winkler C, Laggerbauer B, Fischer U (2014). Identification of a PRPF4 loss-of-function variant that abrogates U4/U6U5 Tri-snRNP integration and is associated with retinitis pigmentosa. PLoS ONE.

[23] Benaglio P, San JP, Avila-Fernandez A, Ascari G, Harper S, Manes G, Ayuso C, Hamel C, Berson EL, Rivolta C (2014). Mutational screening of splicing factor genes in cases with autosomal dominant retinitis pigmentosa. MOL VIS.

[24] Horowitz DS, Kobayashi R, Krainer AR (1997). A new cyclophilin and the human homologues of yeast Prp3 and Prp4 form a complex associated with U4/U6 snRNPs. RNA.

[25] Craige B, Tsao CC, Diener DR, Hou Y, Lechtreck KF, Rosenbaum JL, Witman GB (2010). CEP290 tethers flagellar transition zone microtubules to the membrane and regulates flagellar protein content. J CELL BIOL.

