# Supplementary material for: Clinical and whole exome sequencing findings in children from Yunnan Yi minority ethnic group with retinitis pigmentosa: two case reports
Source: J Med Case Rep. 2023 Jun 2;17:226. doi: 10.1186/s13256-023-03830-3 (PMC10236786; doi:10.1186/s13256-023-03830-3)
Supplement: Supplementary file 1 — Additional file 1: Table S1. The primer sequences. [file 13256_2023_3830_MOESM1_ESM.docx]

Table S1 The primer sequences

| Patient | Forward primer sequence | Reverse primer sequence |
| --- | --- | --- |
| 1 | CTCTCCTCACAGGCCACTTC | GGCCAGCTCACGAGTAAAAA |
| 2 | TTGGTCGAGTTTGGGACCTAC | TAAGCTAAAGCCTGGCCTGC |
